# Does relational mobility vary across national regions? A within-country examination

**DOI:** 10.1371/journal.pone.0235172

**Published:** 2020-07-02

**Authors:** Taciano L. Milfont, Robert Thomson, Masaki Yuki

**Affiliations:** 1 Victoria University of Wellington, Hamilton, New Zealand; 2 Hokusei Gakuen University, Sapporo, Japan; 3 Hokkaido University, Sapporo, Japan; Universitat de Barcelona, SPAIN

## Abstract

Relational mobility is a socio-ecological construct quantifying how much freedom and opportunity a society affords individuals to choose and dispose of interpersonal relationships. Past research has confirmed that relational mobility varies across nations, but no large-scale study has examined the degree to which relational mobility may vary within a single nation. We report two studies (Study 1, *N* = 647; Study 2, *N* = 7343) exploring within-country similarity or variability in relational mobility across all 27 states and five geo-socio-political regions in the continent-size country of Brazil. Results confirmed the measurement equivalence of the Relational Mobility Scale across respondents from all Brazilian states. Notably, relational mobility scores were uniform across Brazilian regions and states, indicating a common national culture regarding the amount of opportunities Brazilians have in selecting new relationship partners within their social context. Replicating existing findings, relational mobility was positively associated with pro-active tendencies that help people retain relationships—levels of intimacy and self-disclosure toward a close friend—indicating that friends tend to feel closer intimacy to their close friends, and reveal serious personal information to a larger degree in social contexts where opportunities to find and retain relationships with like-minded others are greater.

## Introduction

Socio-ecological contexts vary in the degree they afford mobility with regards to interpersonal relationships and group memberships. While high-mobility contexts give individuals the opportunity and freedom to choose new and discard old relationships, individuals in low-mobility contexts have fewer opportunities and less freedom to select interaction partners based on personal preferences. Drawing on this observation, scholars have conceptualised relational mobility as a socio-ecological construct reflecting how much freedom in interpersonal or intergroup choice a given social environment affords individuals [1, 2]. This conceptualization follows a socio-ecological approach to behaviour and psychological tendencies, where those tendencies are viewed as adaptive strategies individuals follow (either consciously or not) in order to adapt to the level of relational mobility in their society.

This conceptualisation warrants objective measurement of relational mobility with archival material or observations, but such measurements have not been attempted to date. Relational mobility has been typically measured with self-report surveys accessing individuals’ perceptions of how much freedom and opportunity their social milieu affords individuals to choose or dispose of interpersonal relationships. Hence, self-report measures of relational mobility assess individuals’ perceptions of the “the amount of opportunities people have to select new relationship partners in a given society or social context” (1 p. 3). Yuki and colleagues [1] developed the Relational Mobility Scale (RMobS) to access individuals’ perceptions regarding how easy or difficult it is for people in their social and ecological setting to enter into, move out of, and form new relationships and group memberships. Notably, the focus is not on measuring how mobile individuals think they are, personally, in choosing relational partners but rather on measuring individuals’ perceptions of how easy it is in general for people in their society to make and change interpersonal relationships.

Using the RMobS, and other proxies for relational mobility, small-scale studies have examined relational mobility both within [3] and across nations (see 2, for a review). The first large-scale study of relational mobility examined variation in relational mobility across 39 societies [4]. Using the RMobS, this study found relational mobility to be higher in North American, Western European and Latin American countries, and lower in East Asian, South Asian, North African, and Middle Eastern countries. Thomson and colleagues [4] also examined predictors and outcomes of relational mobility. They found that relational mobility was lower in societies that both practice settled, interdependent subsistence styles and have stronger ecological and historical threats, and that individuals in societies with higher relational mobility report more pro-active interpersonal behaviours and psychological tendencies that help them acquire and retain relationships.

Although past research has focused mainly on variability in relational mobility across nations, Schug, Yuki and Maddux [5] argued that within-culture variability in relational mobility is likely and consequential, and initial evidence from Yuki et al. (3, Study 2) supports this argument. These authors used regional-average service years (i.e., job tenure, or the average number of years respondents in 11 regions within Japan had worked for their current employer) as an objective indicator of the stability of relationships in the area, and a proxy indicator of relational mobility. They observed variability in job tenure between the regions, and that the positive association between individuals’ self-esteem and happiness was stronger in more relationally mobile regions where people more often move to new workplaces and change their social networks.

Thomson et al.’s [4] large-scale examination of relational mobility corroborate Schug, Yuki and Maddux [5] and Yuki et al.’s (3, Study 2) suggestion that relational mobility could vary within a country. Across the 39 countries and 16,939 people included in their study, only 9% (ICC = 0.09) of variance in relational mobility was accounted for by country of residence. This leaves 91% of variance unaccounted for. However, the degree to which relational mobility varies within a nation has not yet been investigated systematically.

Why are within-country examinations of psychological phenomenon important? Research examining cultural similarities and differences in psychological phenomena has flourished in the last decades with large cross-cultural projects that allow the characterisation of nations based on dimensions of cultural variability. These dimensions include values [6, 7], beliefs [8], and social norms [9], to name but a few. Although these dimensions of cultural variability are useful in describing and contrasting the psychological make-up and behavioural tendencies of individuals from distinct societies, it is possible that national cultures are not uniform within their boundaries. Indeed, an increasing number of studies have used similar large-scale approaches to examine the cultural variability that might exist within single nations. To illustrate, variability in the individualism–collectivism dimension has been observed within the United States [10, 11], and individualism–collectivism variability within China has been liked to specific agricultural practices [12]. When considered as a whole, the United States and China are characterized as individualistic and collectivistic countries, respectively, but these findings provide evidence of within-country variability. Extending relational mobility research, we provide a large-scale examination of relational mobility within Brazil.

## Brazil cultural makeup

Drawing from this growing line of research examining the extent to which dimensions of cultural variability differ within a single national culture [10–12] as well as preliminary evidence that relational mobility may vary within a country [3, 5], we provide the first systematic examination of relational mobility within a single country. We examine the within-country similarity/variability in relational mobility across all federative units (hereafter referred to as states) as well as the five geo-socio-political regions in Brazil, which is a continent-size country both in terms of territory [official territorial area of 8,515,759 km2; 13] and population [207,660,929 inhabitants; 14]. Brazil was colonized by the Portuguese in the 16th century, and six major groups make up the Brazilian population: native peoples (mainly from Tupi and Guarani language groups), Portuguese, Africans, other Europeans, Middle Eastern, and Asian immigrant groups. Brazil is the only Portuguese-speaking nation in the Americas, and its primary religion is Roman Catholic.

Anthropological and psychological accounts indicate great variability of psychological tendencies across states and geo-socio-political areas within Brazil [see, e.g., 15–18]. To illustrate, Torres and colleagues [18] observed that individuals within Brazilian regions with earlier histories of immigration and settlement more strongly endorse values of conformity, tradition and security than individuals in newer settlements in the Center-West region of the country. Hofstede et al. [17] also noted that Masculinity was greater in the North than in the Northeast geo-socio-political regions of the country, which they attributed to the Indigenous and Afro-Brazilian roots of these regions, respectively.

However, there is also evidence indicating homogeneity of Brazilian culture. In contrast to other national cultures, Brazil is often characterized as a collectivistic culture and recent research indicates Brazilian participants display more holistic thinking (i.e., greater attention to context more common in collectivistic societies) than both Chinese and US participants [19]; but Brazil is better described as above average on both Power Distance and Uncertainty Avoidance [6]. Notably, although noting small regional distinctions, Hofstede et al. [17] concluded that their findings provided evidence of a common Brazilian national culture.

Should relational mobility vary across Brazilian regions? On one hand, it is possible that relational mobility is not uniform across the country, considering the large variability in geo-physical and historical contexts between regions within Brazil. In fact, it would be unsurprising to observe variability in psychologies across this diverse and large country in similar ways as has been observed in other large countries such as China and the US [10–12]. Recent studies exploring variability of psychologies within Brazil have documented some distinctions between regions with earlier (versus newer) histories of immigration and settlement [17, 18]. It is thus possible that relational mobility would vary as a function of settlement history and ethnic make-up of the Brazilian geo-socio-political regions. On the other hand, Brazil scored highly on relational mobility as compared with other nations [4], and this high mobility may be reflected in anthropological accounts of Brazilian sociability and hospitality [20]. Existing research also suggests a common Brazilian national culture [6, 15], indicating that Brazilians might perceive the same level of relational mobility in their social context across all regions of the country. We conducted two exploratory studies to examine these possibilities.

## The present study

We report two online studies conducted in Brazil. The research was approved by the Ethics Committee of the Center for Experimental Research in Social Sciences at Hokkaido University. The data were analyzed anonymously. In Study 1 (*N* = 647) we perform secondary analysis of survey data from Brazil previously collected as part of the aforementioned project examining relational mobility in 39 societies [4]. In this first study, we examine the measurement model of the RMobS as well as the variability of relational mobility across the Brazilian geo-socio-political regions.

Study 2 (*N* = 7343) extends the first study in three significant ways. We first examine the measurement invariance of the RMobS across all 27 states in Brazil. Once we established whether participants from all states respond to the scale in statistically comparable ways, we then examine whether scores of relational mobility vary across the Brazilian states and regions, or whether perceptions of relational mobility are the same across this vast country, replicating or not Study 1 findings. Finally, we use multilevel modelling to examine the associations between relational mobility and outcome variables linked to relational mobility. As Thomson et al. [4] reported, individuals within socio-ecological contexts high in relational mobility are more likely to report pro-active interpersonal behaviours as well as psychological tendencies that help them build and retain relationships. They also found that perceived similarity between one’s best friend and oneself is higher in societies that are higher than lower in relational mobility, which can be a relational consequence of high freedom in relational choice. In line of these reasonings, Thomson and colleagues observed a positive association between relational mobility and intimacy, self-disclosure and interpersonal similarity, indicating that relationally mobile societies tend to share personal information more quickly (e.g., self-disclosure) and report higher intimacy and feelings of interpersonal similarity. The final goal of Study 2 is to test whether these cross-national results replicate *within* a nation-state.

## Study 1

### Method

#### Participants and procedure

The World Relationships Study [4] recruited participants using Facebook advertisements in 39 societies (see more information in the S1 File). Societies were selected in order to maximize variation in geography and cultural blocks, as well as Facebook penetration rate to maximize sample diversity within each country. Responses from 499 participants from Brazil were analysed in the Thomson et al.’s [4] study, from a total of 738 participants who clicked past the initial survey landing page. While Thomson et al.’s [4] goal during data cleaning was to minimize inclusion of participants who had dropped out early in the survey, in the current study we were concerned primarily with maximizing the number of participants who had, at a minimum, responded to the RMobS. In the survey flow, this scale was presented to participants on the first page of the survey after the informed consent landing page, while demographic questions, including self-reported state of residence in Brazil, were on the last page of the 6-page survey.

Thomson et al. [4] removed responses with missing information regarding country of residence. Beyond country of residence, our focus is on the state of residence in Brazil, but the state of residence question appeared in the last survey page and this information was missing for 214 participants (out of the 738 participants who clicked past the initial survey landing page). We increased sample size across the Brazilian regions by using an online tool to identify the participants’ missing states from their IP addresses (http://www.bulkseotools.com/bulk-ip-to-location.php).

We removed cases with duplicate IP addresses (retaining the first response on file), those from outside Brazil (as identified by the IP address), cases with more than 33% of data missing for the RMobS, and cases that completed the full survey under three minutes. Because the RMobS is balanced with respect to positively and negatively keyed items, we also removed cases where 80% of responses included only the extreme response values (1, 2, 5 or 6). After these procedures, we had relational mobility data from 647 participants (92% female; *M*_age_ = 22.31, *SD*_age_ = 9.57).

Table 1 presents the distribution of participants for each Brazilian state, ranging from two participants in Roraima (and none in Amapá) to 101 participants in both São Paulo and Minas Gerais. Considering the low sample in most states, we grouped participants to the five geo-socio-political regions of the country: Centre-West (*n* = 61), North (*n* = 36), Northeast (*n* = 160), South (*n* = 131), and Southeast (*n* = 259). We also considered the regrouping of the Federal District within the Southeast region instead of the Centre-West region as suggested by Hofstede et al. [17], but this regrouping had no effect on the results reported in both studies so we report results only when groupings were according to the traditional allocation of the Federal District within the Centre-West region.

**Table 1:** Descriptive statistics of the participants across Brazilian states.

	Study 1				Study 2					
Brazilian sate	Total	*M*age	*SD*age	Female (%)	Friend Target	Romance Target	Total	*M*age	*SD*age	Female (%)
Acre ^a^	5	28.40	14.47	80.0	41	43	84	31.56	15.67	81.8
Alagoas ^b^	9	20.44	7.21	88.9	130	135	265	22.82	8.89	83.3
Amapá ^a^	0	–	–	–	97	89	186	29.23	11.31	80.9
Amazonas ^a^	7	23.17	5.42	83.3	126	99	225	26.78	10.81	87.2
Bahia ^b^	32	25.23	11.18	95.5	187	198	385	23.88	10.25	87.6
Ceara ^b^	38	19.76	6.35	96.6	176	155	331	23.96	13.38	89.5
Distrito Federal ^c^	3	21.33	4.04	66.7	118	193	311	28.42	14.29	85.2
Espírito Santo ^d^	16	18.90	8.58	100	90	148	238	26.67	12.29	80.4
Goiás ^c^	29	20.54	8.44	88.9	146	148	294	26.02	11.36	85.6
Maranhão ^b^	10	21.75	5.57	87.5	103	127	230	26.94	11.26	89.5
Mato Grosso ^c^	16	23.73	8.68	100	93	124	217	28.51	11.80	88.2
Mato Grosso do Sul ^c^	13	20.80	5.16	90.0	93	145	238	29.31	35.55	85
Minas Gerais ^d^	101	23.43	10.32	86.4	217	220	437	24.43	10.29	85.8
Pará ^a^	11	18.11	3.86	100	81	119	200	35.21	14.90	93.3
Paraíba ^b^	15	26.25	13.83	91.7	101	152	253	28.32	13.70	88.9
Paraná ^e^	59	21.29	8.73	100	193	339	532	23.74	11.16	82.8
Pernambuco ^b^	29	22.05	9.46	84.2	168	184	352	23.81	9.35	85.8
Piauí ^b^	7	14.80	2.95	80.0	82	118	200	27.08	10.67	89.7
Rio de Janeiro ^d^	41	24.76	12.12	90.9	129	191	320	26.30	13.68	84.5
Rio Grande do Norte ^b^	15	20.45	6.52	100	106	123	229	24.51	10.16	82.3
Rio Grande do Sul ^e^	45	20.80	8.80	97.2	125	161	286	25.60	12.13	85.4
Rondônia ^a^	8	21.50	4.37	100.0	90	123	213	28.68	12.86	85.6
Roraima ^a^	2	16.50	3.54	100	63	51	114	26.70	8.83	83.6
Santa Catarina ^e^	27	20.33	5.83	90.5	104	144	248	24.51	9.57	85.7
São Paulo ^d^	101	24.49	11.64	90.4	262	298	560	26.43	14.01	84.5
Sergipe ^b^	5	14.75	3.10	100	105	103	208	26.48	10.50	84
Tocantins ^a^	3	18.00	6.00	100	90	97	187	29.69	13.19	83.6

“Friend Target” refers to number of participants for whom target of dependent variables was their best friend, “Romance Target” refers to number of participants for whom target of dependent variables was their romantic partner. Superscripts for the Brazilian states indicate their respective geo-socio-political region in the ^a^ North (*k* = 7)

^b^ Northeast (*k* = 9)

^c^ Centre-West (*k* = 4)

^d^ South (*k* = 3) or

^e^ Southeast (*k* = 4).

#### Measures

Yuki and colleagues [1] developed the 12-item RMobS to access individuals’ perceptions of the degree to which people in a society or social context have the freedom and opportunity to choose and dispose of relationships based on personal preference. The scale includes five items for a “meeting” factor capturing the degree to which a society or social context affords opportunities for individuals to meet new people and forge new relationships (e.g., “They (the people around you) have many chances to get to know other people”), and seven items for a “choosing” factor capturing the degree to which people have the freedom to choose and leave relationships based on personal preference (e.g., “They (the people around you) are able to choose, according to their own preferences, the people whom they interact with in their daily life”). Items are rated on a 6-point agreement scale anchored by 1 (*strongly disagree*) and 6 (*strongly agree*).

Thomson et al. [4] used a translation/back-translation procedure to translate the English version of the survey into each of the other 19 languages. Separate professional translators first translated and back-translated surveys, and then English-bilingual collaborators for whom the target language was their native tongue double checked both translations and approved of the final version. The Brazilian Portuguese version of the RMobS is available here: https://osf.io/e5hm9/.

### Results and discussion

**Fig 1:**
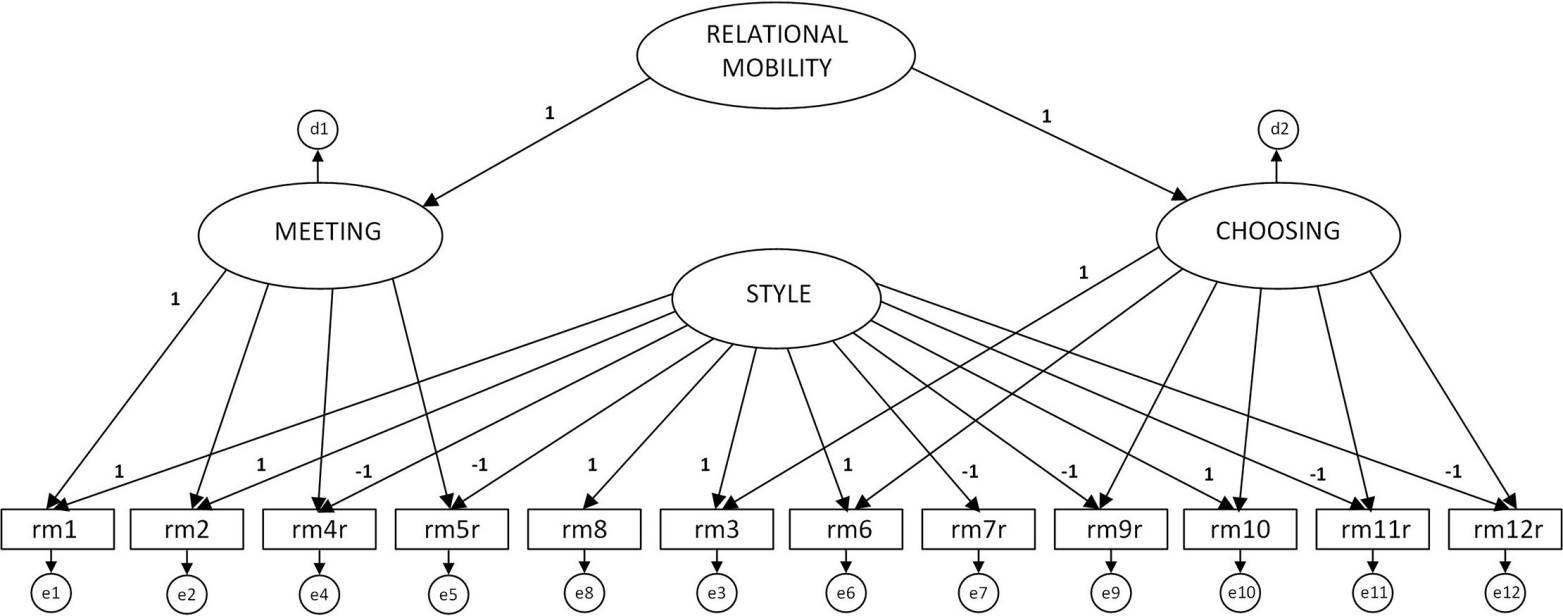
Measurement model of the relational mobility scale with two first-order content factors, one second-order content factor, and one common-method bias factor modeling acquiescence responding style.

Fig 1 depicts the measurement model of the RMobS that has been tested and validated cross-nationally by Thomson et al. [4]. The model includes four latent factors: two first-order content factors, one second-order content factor, and one common-method bias factor modeling acquiescent response style. Instead of focusing on the sub-dimensions of relational mobility of “meeting” and “choosing”, this model focuses on an overall latent construct.

We were unable to run a multi-group confirmatory factor analysis to test the measurement invariance of this model across participants clustered in states or regions given the complexity of the model and small sample sizes or regions. We provide a thorough examination of this issue in Study 2. Here we only examined the fit of the model across all participants with robust maximum likelihood estimation in Mplus (version 7.4). The model had good fit to the data: S-B χ^2^ (50) = 93.845, *p* < .001, *CFI = .951, *SRMR = .040, *RMSEA = .037 (90% *CI* = .025, .048). The average standardized loading was .478 and the lowest was for Item 9 in the “choosing” factor, which was still statistically significant (*b* = .276, *p* < .001).

The factor scores generated from this model and the raw scores (averaging over the items after reversing coding the relevant items) were highly correlated (*r* = .980, *p* < .001; *N* = 646). We used the factor scores to examine whether relational mobility varied across the five geo-socio-political regions. A one-way ANOVA showed that the factor scores did not vary statistically across the regions (*F* = .139, *p* = .968). This lack of variability is evidenced by the respective raw and factor scores aggregated for each region: North (4.340, .00036), Northeast (4.419, .02019), Centre-West (4.353, -.01867), South (4.355, -.00690), and Southeast (4.365, -.00457).

## Study 2

The results from Study 1 suggest a common social reality within Brazilian regions regarding relational mobility. The small sample sizes across the geo-socio-political regions reduced our confidence in the findings, however. To overcome this shortcoming, we examined whether the observed uniformity in relational mobility replicates in a more systematic investigation by examining possible variations not only in the five broader Brazilian regions but also across all 27 states. Thus, Study 2 examines the measurement invariance of the RMobS across all 27 states in Brazil, whether scores of relational mobility vary or not across the Brazilian states and regions, and associations between relational mobility and outcome variables.

### Method

#### Participants and procedure

We used the same Facebook advertisement strategy to recruit participants as in Thomson et al. [4], but this time the explicit focus was on recruiting participants from all states in Brazil. Since Internet penetration (and therefore Facebook usage) varies across states, we used three survey waves to maximize sample sizes in all geographic locations. Wave 1 was collected in Oct/Nov 2015 (*N* = 3688; 83.5% female; *M*_age_ = 20.94, *SD*_age_ = 6.82), Wave 2 in Jan/Feb 2016 (*N* = 2522; 90.5% female; *M*_age_ = 31.23, *SD*_age_ = 16.47), and Wave 3 in Sep/Oct 2017 (*N* = 1133; 82.9% female; *M*_age_ = 37.48, *SD*_age_ = 15.88). These numbers represent the final sample for each wave after exclusions. Similar to the procedures used in Study 1, we removed cases with duplicate IP addresses or those from outside Brazil, cases with more than 33% of data missing for the RMobS, cases where 80% of responses included only one of the extreme response values (1, 2, 5 or 6), and participants who completed the survey under 3 minutes.

The final sample comprised 7343 participants (85.6% female; *M*_age_ = 26.30, *SD*_age_ = 13.53) from all Brazilian states. We used the same online tool as in Study 1 to increase sample size across the Brazilian for participants with missing information on their state of residence (*n* = 2051). Sample sizes per state varied from 84 (Acre) to 560 (São Paulo; see Table 1), and comprised 1000+ participants in each region: Centre-West (*n* = 1060), North (*n* = 1209), Northeast (*n* = 2453), South (*n* = 1066), and Southeast (*n* = 1555).

#### Measures

Study 2 examines relational mobility plus the three measures detailed below (see S1 File for additional measures not examined here). Items of all four measures targeted either a participant’s “closest friend” or “romantic partner”. Advertisements focusing on either target were randomly presented to Facebook users targeted by Brazilian state. A roughly equal number of participants was obtained for each version of the survey.

*Relational mobility*. The same 12-item RMobS described in Study 1 was used in this study. Items are rated on a 6-point agreement scale anchored by 1 (*strongly disagree*) and 6 (*strongly agree*). Relational mobility scores were statistically higher for participants who responded to the friendship version of the survey (*N* = 4,027, *M* = 4.47, *SD* = .65) than for those who responded to the romance version (*N* = 3,316, *M* = 4.40, *SD* = .65), *t*(7341) = 4.81, *p* < .001, *d* = .11. Considering that this difference was small, that Thomson et al. [4] did not observe such a difference in their 39-nation study, and that exploration of this difference is outside the scope of our study, we did not investigate this further. We thus collapsed the responses to the two versions of the survey to examine how general perceptions of relational mobility impacts intimacy, self-disclosure and interpersonal similarity for both a closest friend and romantic partner.

*Intimacy*. We used a 10-item version of Sternberg’s triangular love scale to assess intimacy [21]. Some example items in the scale are “I receive considerable emotional support from [friend/romantic partner]”, and “I feel that [friend/romantic partner] really understands me”. Items were rated on a 5-point agreement scale anchored by 1 (*strongly disagree*) and 5 (*strongly agree*), and Cronbach’s alphas were .92 and .91 for the friendship and romance versions, respectively.

*Self-disclosure*. We used five items to measure self-disclosure [5]. Participants were asked to indicate the degree to which they have revealed themselves to their friend/romantic partner in term of secrets, embarrassing experiences, failures, worries, and bad things that had happened to them. Items were answered on a 5-point scale anchored by 1 (*I have not revealed any information at all*) to 5 (*I have revealed even the most serious information*), and Cronbach’s alphas were .88 for both survey versions.

*Interpersonal similarity*. Participants were asked to rate how similar they are with their friend/romantic partner in five aspects: personality, hobbies, values, behaviours, and lifestyles [22]. Items were rated on a 7-point scale anchored by 1 (*not similar at all*) and 7 (*very similar*), and Cronbach’s alphas were .83 and .81 for the friendship and romance versions, respectively.

### Results and discussion

#### Measurement invariance of measures

We started by examining whether participants from all Brazilian states used the four measures in the same way. We compared the fit of the model in Fig 1 against alternative models, and then ran multi-group analysis to confirm configural, metric, and scalar invariance of the RMobS. Table 2 presents the results of all nine models tested. In brief, the fit statistics support the measurement model depicted in Fig 1 across all participants and confirms the measurement equivalence of the RMobS across participants from all Brazilian states. The model also had good fit across the Brazilian regions: S-B χ2 (333) = 851.52, *p* < .001, *CFI = .947, *SRMR = .036, *RMSEA = .033 (90% *CI* = .030, .035).

**Table 2:** Fit statistics for measurement models and measurement invariance testing of the relational mobility scale in Study 2.

Model	*S-B χ^2^	*df*	*CFI	*SRMR	*RMSEA	*RMSEA 90% CI	Model Comparison	Δ*CFI	Δ*SRMR	Δ*RMSEA	Decision
Model 1 ^a^	514.227	50	.952	.026	.036	.033, .038	–	–	–	–	–
Second-order model (Fig 1)											
Model 1a ^a^	2001.275	60	.872	.045	.066	.064, .069					
Second-order model with another score for acquiescence											
Model 1b ^a^^,^ ^b^	2709.662	51	.725	.071	.084	.082, .087	–	–	–	–	–
Second-order model with no STYLE factor											
Model 2	1971.378	1350	.939	.052	.041	.037, .045	–	–	–	–	–
Configural invariance											
Model 2a ^c^	1972.929	1353	.939	.052	.041	.037, .045	–	–	–	–	–
Configural invariance, constraining residual variance of the CHOOSING factor to 0 in 3 states. All subsequent models include these constrains											
Model 3	2251.457	1612	.937	.067	.038	.034, .042	3 vs. 2a	-.002	.015	-.003	Accept
First-order factor loadings invariant											
Model 4	2565.876	1846	.929	.070	.038	.034, .041	–	–	–	–	–
Observed variable intercepts invariant											
Model 4a	2565.604	1849	.930	.070	.038	.034, .041	4a vs. 3	-.007	< .001	< .001	Accept
Observed variable intercepts invariant, constraining residual variance of the CHOOSING factor to 0 in 3 states. All subsequent models include these constrains											
Model 5	2582.519	1875	.931	0.70	.037	.034, .041	5 vs 4	.001	< .001	< .001	Accept
First-order latent variable intercepts invariant											
Model 6	2617.441	1901	.930	.074	.037	.034, .041	6 vs. 5	-.001	< .001	< .001	Accept
Second-order factor variance invariant											

Asterisks denote robust fit indices.

^a^ Models 1, 1a and 1b had more parameters than the number of clusters, so the standard errors of the model parameter estimates may not be trustworthy.

^b^ Model 1b had another latent factor, N_AGREE, measured by a single observed acquiescent response style variable created by a simple summation of the frequency an individual responded in the affirmative to a number of semantically similar but oppositely keyed items in the survey. This latent factor was highly correlated with the STYLE factor (*r* = .805, *p* < .001), demonstrating that the latent “style” factor in our measurement model is indeed measuring acquiescent response style.

^c^ Model 2a: countries with residual variance of the CHOOSING factor constrained to 0 were Acre, Paraíba and Piauí. Model 4a: countries with residual variance of the CHOOSING factor constrained to 0 were Goiás, Rondônia and São Paulo.

Examination of the psychometric properties of the other three scales confirmed the one-factor structure and good loadings of the scale items (see Table 3). The results also confirmed the configural, metric, and scalar invariance of the measures across participants from all 27 Brazilian states irrespective of social target (see Table 4).

**Table 3:** Factor loadings and model fit for the self-disclosure, similarity, and intimacy scales in study 2 within a pooled culture (State)-free sample.

Target	Latent variable	Standardized factor loadings (latent variable → observed variable *x*)										Fit Indices					
		1	2	3	4	5	6	7	8	9	10	S-B χ^2^	*df*	*CFI	*SRMR	*RMSEA	(90% CI)
Close friend	Disclosure ^a^	.783	.795	.798	.742	.728						51.823	4	.989	.016	.060	(.046, .075)
	Similarity ^b^	.666	.621	.659	.758	.711						72.191	5	.983	.020	.064	(.052, .078)
	Intimacy ^c^	.756	.758	.609	.786	.655	.803	.621	.705	.693	.621	288.654	33	.962	.026	.048	(.043, .053)
Romantic partner	Disclosure ^d^	.773	.795	.816	.727	.713						36.281	4	.991	.012	.058	(.042, .076)
	Similarity ^e^	.659	.645	.715	.753	.729						45.675	5	.984	.020	.059	(.044, .075)
	Intimacy ^f^	.832	.802	.542	.812	.626	.801	.771	.766	.770	.627	298.162	34	.973	.027	.056	(.050, .062)

^a^ *N* = 2,411.

^b^ *N* = 2,362.

^c^ *N* = 2,501.

^d^ *N* = 3,322.

^e^ *N* = 3,236.

^f^ *N* = 3,422. Error covariances allowed: Disclosure items 4 with 5 (both targets), Intimacy items 3 with 5 (close friend target) and items 3 with 5 and items 1 with 9 (romantic partner target). The models for the intimacy scale had more parameters than the number of clusters, so the standard errors of the model parameter estimates may not be trustworthy.

**Table 4:** Measurement invariance indices from multi-group confirmatory factor analysis for disclosure, similarity, and intimacy scales, analyzed by target, in Study 2.

Model	Target	Scale	S-B χ^2^	*df*	*CFI	*SRMR	*RMSEA	*RMSEA 90% CI	Comparison ^a^	Δ*CFI	Δ*SRMR	Δ*RMSEA	Decision
**Model 1**	Close friend	Disclosure	214.900	108	.977	.031	.090	(.072, .107)	-	-	-	-	Accept
Configural invariance		Similarity	194.265	135	.982	.036	.061	(.040, .079)	-	-	-	-	Accept
		Intimacy	1626.879	918	.928	.055	.078	(.072, .084)	-	-	-	-	Accept
	Romantic partner	Disclosure	132.342	108	.994	.025	.050	(< .001, .077)	-	-	-	-	Accept
		Similarity	225.933	135	.970	.040	.088	(.067, .107)	-	-	-	-	Accept
		Intimacy	1327.116	891	.956	.049	.073	(.064, .081)	-	-	-	-	Accept
**Model 2**	Close friend	Disclosure	327.475	212	.975	.075	.067	(.052, .080)	2 vs 1	-.002	.044	-.023	Reject
Metric invariance		Partial ^b^	264.716	160	.977	.054	.073	(.057, .088)	2a vs 1	< .001	.023	-.017	Accept
		Similarity	337.669	239	.967	.080	.069	(.051, .085)	2 vs 1	-.015	.044	.008	Reject
		Partial ^c^	278.509	213	.980	.066	.051	(.032, .066)	2a vs 1	-.002	.030	-.010	Accept
		Intimacy	1912.720	1152	.923	.166	.072	(.066, .078)	2 vs 1	.010	.118	-.007	Reject
		Partial ^d^							2a vs 1				Accept
	Romantic partner	Disclosure	244.082	212	.992	.073	.041	(< .001, .063)	2 vs 1	-.002	.048	-.009	Reject
		Partial ^e^	184.219	169	.994	.051	.041	(< .001, .066)	2a vs 1	< .001	.026	-.009	Accept
		Similarity	337.669	239	.967	.080	.069	(.051, .085)	2 vs 1	-.003	.040	-.019	Reject
		Partial ^f^	280.869	187	.969	.060	.076	(.057, .093)	2a vs 1	-.001	.020	-.012	Accept
		Intimacy	1575.573	1125	.955	.103	.066	(.058, .073)	2 vs 1	-.001	.054	-.007	Reject
		Partial ^g^	1382.203	943	.956	.060	.071	(.063, .079)	2a vs 1	< .001	.011	-.002	Accept
**Model 3**	Close friend	Disclosure	398.945	264	.971	.063	.064	(.051, .077)	3 vs 2a	.006	.009	-.009	Accept
Scalar invariance		Similarity	419.818	317	.969	.076	.052	(.038, .065)	3 vs 2a	-.011	.010	.001	Accept
		Intimacy	2051.674	1204	.914	.084	.075	(.069, .080)	3 vs 2a	-.014	.007	-.001	Reject
		Partial ^j^	1790.018	1048	.925	.080	.075	(.069, .081)	3a vs 2a	-.003	.003	-.001	Accept
	Romantic partner	Disclosure	295.762	264	.992	.065	.037	(< .001, .058)	3 vs 2a	-.002	.014	-.004	Accept
		Similarity	379.700	291	.970	.067	.059	(.041, .075)	3 vs 2a	.001	.007	-.017	Accept
		Intimacy	1686.450	1177	.949	.067	.068	(.061, .076)	3 vs 2a	-.008	.006	-.003	Accept

^a^ Fit index value comparisons are with the immediately preceding model’s respective scale, e.g., Model 3 disclosure (target: close friend) vs. Model 2 disclosure (target: close friend).

^b^ Factor loading equality constraints relaxed: Items 1 and 4.

^c^ Factor loading equality constraints relaxed: Items 1 and 3.

^d^ Factor loading equality constraints relaxed: Items 1, 3, 4, 5, 6, 7 and 8.

^e^ Factor loading equality constraints relaxed: Items 1 and 4.

^f^ Factor loading equality constraints relaxed: Items 1 and 3.

^g^ Factor loading equality constraints relaxed: Items 1, 3, 4, 5, 6, 7 and 8.

^j^ Intercept constraints relaxed: Items1, 2, 3, 5, 8 and 9.

#### Variability of relational mobility across Brazilian states and regions

We used the factor scores and latent means (from Model 6 in Table 5) to extend the results of Study 1 and examine variability of relational mobile across the Brazilian states and the five geo-socio-political regions. As in Study 1, the factor scores and the raw scores were highly correlated (*r* = .977, *p* < .001, *N* = 7343).

**Table 5:** Factor loadings of the relational mobility scale in Study 2.

Item label	Factor	Factor loading
rm1	MEETING	.565
rm2	MEETING	.439
rm3	CHOOSING	.508
rm4r	MEETING	.332
rm5r	MEETING	.530
rm6	CHOOSING	.408
rm7r	CHOOSING	.389
rm8	MEETING	.497
rm9r	CHOOSING	.335
rm10	CHOOSING	.513
rm11r	CHOOSING	.448
rm12r	CHOOSING	.509

Factor loadings are standardized estimates (STDYX Standardization) from Model 1 in Table 2. English and Brazilian Portuguese wording of the items are available here: https://osf.io/e5hm9/.

Starting with the state comparisons, the results indicated that less than 1% of the variability in scores of the RMobS are attributed to variability across the Brazilian states (ICC = .009). A one-way analysis of variance (ANOVA) on participants’ relational mobility latent factor scores was statistically significant, as expected given the large sample size, but the effect size was very small, *F*(26, 7316) = 3.32, *p* < .001, *η*^2^ = .012. Similarly, a one-way analysis of variance (ANOVA) on participants’ relational mobility latent scores across the five geo-socio-geographic regions indicated a statistically significant but very small effect, *F*(4, 7338) = 3.78, *p* = .005, *η*^2^ = .022. The lack of variability is evidenced by the respective factor and raw scores of relational mobility aggregated for each state and region reported in Table 6 (see also S1 File for a visual inspection). Hence, these results indicate homogeneity in relational mobility across regions and states in Brazil.

**Table 6:** State-level scores of relational mobility in Study 2.

State	Raw Score	Factor Score
Acre ^a^	4.411	.00575
Alagoas ^b^	4.379	-.00895
Amapá ^a^	4.427	-.00344
Amazonas ^a^	4.397	-.00323
Bahia ^b^	4.481	.06091
Ceara ^b^	4.466	.04451
Distrito Federal ^c^	4.436	.01395
Espírito Santo ^d^	4.490	.05708
Goiás ^c^	4.431	-.00140
Maranhão ^b^	4.535	.10415
Mato Grosso ^c^	4.409	-.02239
Mato Grosso do Sul ^c^	4.437	.01274
Minas Gerais ^d^	4.440	.01527
Pará ^a^	4.431	.01535
Paraíba ^b^	4.330	-.08123
Paraná ^e^	4.424	.00031
Pernambuco ^b^	4.481	.06412
Piauí ^b^	4.419	.00283
Rio de Janeiro ^d^	4.452	.03464
Rio Grande do Norte ^b^	4.412	.00494
Rio Grande do Sul ^e^	4.449	.02579
Rondônia ^a^	4.321	-.09392
Roraima ^a^	4.453	.02661
Santa Catarina ^e^	4.371	-.04243
São Paulo ^d^	4.462	.02511
Sergipe ^b^	4.458	.02424
Tocantins ^a^	4.466	.02028

The factor scores were generated from Model 6 in Table 2 and the raw scores were computed by averaging over the items after reversing coding the relevant items; these scores were then aggregated at the state level. Superscripts for the Brazilian states indicate their respective geo-socio-political region in the ^a^ North (k = 7), ^b^ Northeast (k = 9), ^c^ Centre-West (k = 4), ^d^ South (k = 3) or ^e^ Southeast (k = 4). The factor and raw scores for the regions were, respectively: North (4.411, -.00909), Northeast (4.444, .02793), Centre-West (4.429, .00198), South (4.418, -.00280) and Southeast (4.458, .02920). The factor and raw scores for the regions observed in Study 1 are reported in text.

#### Relational mobility and outcome variables

Results from both studies indicate low variability in relational mobility scores in Brazil. At the same time, the conceptualization and measurement of relational mobility as a socio‐ecological construct implies that individuals’ perceptions of relational mobility in their surroundings (a contextual, level‐2 variable) can have downstream consequences on individual difference measures (level‐1 variables). We thus employed multilevel analyses to examine the associations between relational mobility (modelled using latent means at the state level), and intimacy, self-disclosure and interpersonal similarity (modelled at the individual level). We included gender, age, and household socio-economic level as individual-level control variables. Excluding these variables from the models did not alter the main findings and interpretations, and in most cases, the coefficients were stronger when these variables were excluded (all results with and without covariates, as well as the model syntax and output, are available here: https://osf.io/9xuvy/).

Table 7 presents the results of multilevel analyses for each target at the state level. Latent mean levels of relational mobility at the state level were significantly and positively associated with self-disclosure, similarity and intimacy when the target was a close friend. No reliable associations were observed for state-level relational mobility and these variables when the target was a romantic partner. This is unsurprising since relational mobility is conceptually more relevant for friendship than romantic relationships [2], and Thomson et al. [4] also observed stronger associations between country-level relational mobility and all three measures when the target was a close friend than when it was a romantic partner. Our results thus replicate these findings across states within a single country.

**Table 7:** Multi-level analyses predicting interpersonal behavior and psychology from state-level relational mobility in Study 2.

Target	Level-1 Dependent Variable	Model^c^	Dependent Intercept γ_00_ (SE)	Within-group Variance *r* (SE)	Between-group Variance *u*_0_ (SE)	Level-2 Predictor
						Relational Mobility (SE) γ_01_
Close friend^a^	Self-disclosure	1	-.701*** (.097)	.527*** (.019)	< .001 (.002)	—
		2	-.651*** (.096)	.526*** (.019)	.004 (.002)	.261* (.116)
	Similarity	1	-.856*** (.092)	1.132*** (.044)	.009 (.005)	—
		2	-.818*** (.097)	1.130*** (.044)	.010 (.007)	.570* (.280)
	Intimacy	1	-.386*** (.071)	.312*** (.014)	.001 (.001)	—
		2	-.386*** (.071)	.311*** (.013)	.001 (.001)	.302* (.121)
Romantic partner^b^	Self-disclosure	1	-.530*** (.129)	0.790*** (.023)	0.002 (.003)	—
		2	-.471*** (.135)	0.788*** (.023)	0.003 (.009)	-.283 (.208)
	Similarity	1	-1.207*** (.329)	2.748*** (.114)	0.050* (.020)	—
		2	-1.088** (.338)	2.746*** (.115)	0.042 (.026)	-.647^†^ (.362)
	Intimacy	1	-1.283* (.523)	4.574*** (.193)	0.024 (.016)	—
		2	-1.223* (.527)	4.574*** (.192)	0.023 (.038)	.376 (.435)

^a^ *N* = 3,042, *k* = 27.

^b^ *N* = 2,196, *k* = 27. Model 1: Unconditional means model (includes gender, age and household income as covariates at the individual level); Model 2: Regression with means-as-outcomes with relational mobility on the dependent variables (includes gender, age and household income as covariates at the individual level). Individuals who indicated being older than 80 years old were not included in these analyses.

***p < .001

**p < .01

*p < .05

^†^*p* < .10.

## General discussion

Relational mobility is a socio-ecological construct quantifying how much freedom and opportunity a society affords individuals to choose and dispose of interpersonal relationships, and a large-scale study recently showed meaningful variability in relational mobility across 39 societies [4]. We reported two exploratory studies examining variability in relational mobility within Brazil, which is a continent-size country consisting of 27 geographically and culturally distinct states and 5 geo-socio-political regions.

First, results from both studies confirmed the construct validity of the RMobS––a 12-item measure accessing individuals’ perceptions of how easy or difficult it is for people in their social and ecological setting to enter into, move out of, and form new relationships [1]. Moreover, results from Study 2 confirmed the measurement equivalence of the measure for participants across all 27 Brazilian states. Together with the cross-cultural findings reported by Thomson et al. [4], the results from this study provide further evidence of the psychometric properties of the scale as well as its within-culture validity.

Second, Thomson et al. [4] observed that levels of intimacy, similarity and self-disclosure toward a close friend were higher in relationally mobile societies. Study 2 replicated these findings by showing a positive association between these three pro-active tendencies that help people retain relationships and relational mobility in Brazil. Taken together, these findings support hypotheses regarding the psychological consequences of relational mobility [see 23]: friends tend to feel closer intimacy, more similar to their close friends, and reveal serious personal information to a larger degree in social contexts where opportunities to find desirable and terminate undesirable relationships are greater.

Notably, the main goal of our study was to explore variability in relational mobility within Brazil. Overall our results confirmed the very high degree of individual-level variability observed by Thomson et al. [4] and did not find any meaningful variance within Brazil. Indeed, our results indicate that relational mobility does not vary across regions (Studies 1 and 2) or states (Study 2) in Brazil. This reveals that our participants’ perceptions of the amount of opportunities Brazilians have to select new relationship partners within their social context are fairly uniform across Brazil, and are not dependent on one’s residence in any particular state or region.

This uniformity is consistent with anthropological and sociological work arguing that despite decades of migration in Brazil, a single civic and political entity emerged, which has eventually lead to cultural uniformity and a unitary national conscience [16, 24]. It is thus possible to argue for a common Brazilian national culture where states in Brazil are “much more similar to each other than to Latin American countries, let alone countries worldwide” (18 p. 347). Our findings provide support for this view by showing that the common Brazilian national culture also includes abundant freedom and opportunity afforded to Brazilians to choose and dispose of interpersonal relationships, which might explain their noted sociability and hospitality [20].

As indicated in Fig 1, relational mobility theoretically comprises the two interrelated subcomponents of “meeting” and “choosing”. The interplay and relationships between the two subcomponents are well outside the scope of the current paper but, following a reviewer’s request, we conducted the same analyses reported in Table 6 for the subcomponents (see S1 File). When split into its constituent subcomponents, only the choosing factor shows consistent and statistically significant associations with intimacy, self-disclosure and interpersonal similarity. In line with findings regarding the higher-order relational mobility factor, this is only the case for dependent variables where the target is a close friend. Moreover, the finding of a statistically significant association between the choosing factor (but not the meeting factor) and intimacy is consistent with results found by Yamada, Kito and Yuki [25]. These findings should be explored in future studies to tease out the dynamics of *choice* and *opportunity* in relational mobility’s role in interpersonal behavior.

It is worth noting the gender distribution in our study was heavily skewed towards women (92% and 85.6% female participants in Study 1 and 2). We believe this reflects self-selection bias in the sampling strategy used with Facebook advertisement as well as the survey focus on close interpersonal relationships. The unbalanced gender distribution was similar to the 39-nation study by Thomson et al. [4] where 86.2% of the sample were women, but their analyses showed that gender explained less than .05% the variance in relational mobility scores. We control for gender in the critical analyses reported in Table 6, but future studies should seek a better gender distribution in their sampling. Finally, we used an online tool to identify the participants’ missing states from their IP addresses in order to increase the sample size across the Brazilian regions (representing 28.9% and 27.9% of the final samples in Studies 1 and 2, respectively). Although we believe this was a reasonable strategy, it is possible that some participants masked their correct IP location when completing the survey and/or that the tool did not accurately identified the state location of some participants. Future studies should replicate our findings across all Brazilian states with a better measure of participants’ actual location.

To conclude, the present study provides the first systematic within-nation examination of relational mobility. The findings indicate very high degree of individual-level variability and very low degree of within-country variance in relational mobility in Brazil. Hence, our findings provide support for a common Brazilian national culture regarding relational mobility. Considering the links of relational mobility with historical variations in subsistence styles and ecological threats [4], thinking styles [26], and faster spread of infectious viruses [27], we hope this study will motivate further relational mobility research within and across nations.

## Supporting information

S1 File (DOCX)
