# Supplementary material for: Does relational mobility vary across national regions? A within-country examination
Source: PLoS One. 2020 Jul 2;15(7):e0235172. doi: 10.1371/journal.pone.0235172 (PMC7332055; doi:10.1371/journal.pone.0235172)
Supplement: S1 File — (DOCX) [file pone.0235172.s001.docx]

**Facebook Adds**

We recruited participants to the online survey using Facebook advertisements, displayed

to logged-in Facebook users on the site**.** Fig S1 and Fig S2 present the advertisements shown to Brazilians promoting the survey targeting either a participant’s “closest friend” (the Friendship Survey) or “romantic partner” (the Romance Survey), respectively.

These advertisements were created Thomson et al. (2018) and are available under a CC-BY license (friend version: <https://osf.io/a47u5/>; romance version: <https://osf.io/f47xd/>). The images were created using Adobe Illustrator and Adobe Photoshop. The actual illustrations were created for us in the original multi-country paper (Thomson et al., 2018) by an illustrator whom we paid to create them; we own the rights to the illustrations, and, as above, have made them available under a CC-BY license. For the Brazil study reported in Study 2 of the main article, we modified the original images slightly, by adding the 'you can do this on your smartphone' text (again, using Adobe Illustrator).

**
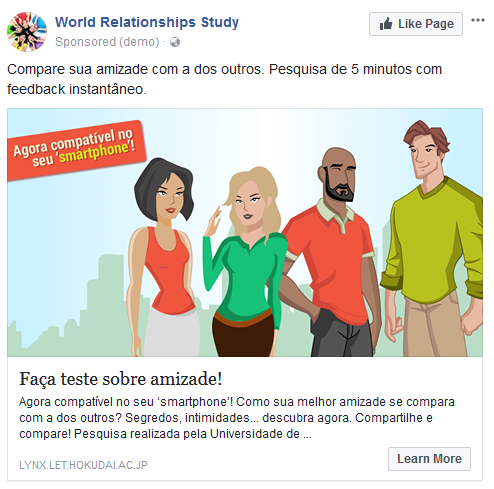
**

**Fig S1. Facebook Advertisement used in Brazil for the Friendship Survey**

**
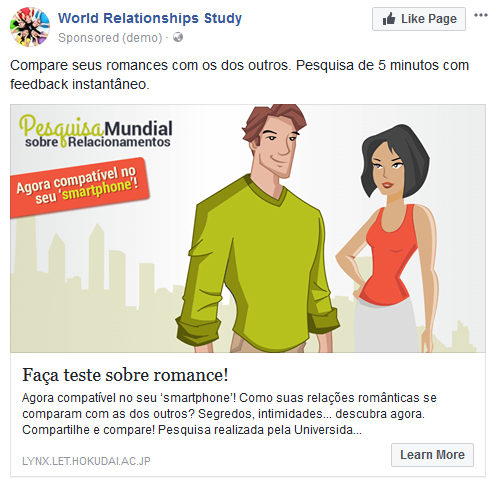
**

**Fig S2. Facebook Advertisement used in Brazil for the Romance Survey**

**Additional Measures**

Our main analyses for Study 2 focused on the three measures that were included in all three waves and that have shown to be consequential to relational mobility (Thomson et al., 2018). However, the Study 2 surveys included other measures not reported in the main text.

The survey included a measure assessing participants’ judgements of the quality of the natural environment in Brazil in Wave 2, and self-esteem and empathy measures in Wave 3. The first measure is not relevant to the present research and while self-esteem and empathy might be related to relational mobility (Newman et al., 2017, Study 4), the small sample sizes across the Brazilian states in Wave 3 restrict proper examination of their relationships with relational mobility.

Besides the three measures described above, we also included four variables that are conceptually related to relational mobility (Thomson et al., 2018). We measured: (1) the number of new acquaintances participants made in the last month, (2) the number of romantic partners the respondents have had, (3) the number of times participants had a romantic partner poached by another person, and (4) the number of times participants have stolen a romantic partner from another person. Thomson et al. (2018) only considered the first three questions in their analyses. As reported in Table S7 of their Supplementary Material, only number of romantic partners was associated with relational mobility. Their findings indicated that the number of romantic partners respondents reported they have had was higher in more relationally mobile regions.

For the sake of completeness, we examined the associations between state-level latent means of relational mobility and the three main variables that reflect opportunity and freedom for relationships in societies. The multilevel model did not converge when adding the partner poached question, which was excluded from the analyses. The multilevel results are presented in Table S1. Relational mobility was only significantly associated with number of romantic partners stolen. This finding suggests that the number of times respondents reported they have stolen a romantic partner from another person was higher in more relationally mobile regions, which seems contra intuitive.

Overall, the results reported by Thomson et al. (2018, Table S7) and our own findings indicate that convergent validity of relational mobility must be further investigated in future studies. It would be productive to expand the list of within-survey self-reported interpersonal variables and potentially include observed measures (when viable) in within- and cross-cultural studies.

**Table S1. Multi-Level Analyses Examining Convergent Validity with Within-Survey Interpersonal Variables (Study 2).**

|  | Level-1 Dependent Variable | Model ^a^ | Dependent Intercept  γ_00_  (SE) | Within-group Variance  *r*  (SE) | Between-group Variance  *u*_0_  (SE) | Level-2 Predictor |
| --- | --- | --- | --- | --- | --- | --- |
|  |  |  |  |  |  | Relational Mobility  (SE) γ_01_ |
|  | New acquaintances in the last month (log) | 1 | .644^***^  (.038) | .165^***^  (.003) | .001^*^  (.001) | — |
|  |  | 2 | .644^***^  (.037) | .165^***^  (.003) | .001^*^  (<.000) | -.007  (.100) |
|  | Number of romantic partners (log) | 1 | .612^***^  (.063) | .071^***^  (.039) | <.000  (<.000) | — |
|  |  | 2 | .622^***^  (.037) | .071^***^  (.002) | <.001  (<.001) | -0.014  (.079) |
|  | Number of times have stolen a romantic partner (log) | 1 | .140^**^  (.042) | .039^***^  (.002) | <.000  (<.000) | — |
|  |  | 2 | .118^**^  (.042) | .038^***^  (.002) | <.000  (<.000) | -0.107^*^  (.042) |

Notes. *N* = 5,128, *k* = 27. Model 1: Unconditional means model (includes gender, age and household income as covariates at the individual level); Model 2: Regression with means-as-outcomes with relational mobility on the dependent variables (includes gender, age and household income as covariates at the individual level). Individuals who indicated being older than 80 years old were not included in these analyses.

^***^*p* < 0.001, ^**^*p* < 0.01, ^*^*p* < 0.05.

**Variability Across Brazilian States**

As reported in the main document, a one-way analysis of variance (ANOVA) on participants’ relational mobility latent factor scores was statistically significant but the effect size was very small, *F*(26, 7316) = 3.32, *p* < .001, *η*2 = .012, indicating homogeneity in relational mobility across states in Brazil. Given the exploratory focus of our studies, we decided to inspect scores visually. As the data did not meet the homogeneity of variances assumption, we employed the Games-Howell post-hoc test to compare states. Fig S3 depicts the state-level differences.

In short, relational mobility scores for respondents in 14 states (Acre, Alagoas, Amapá, Amazonas, Distrito Federal, Goiás, Mato Grosso do Sul, Minas Gerais, Pará, Piauí, Rio Grande do Norte, Roraima, Sergipe, and Tocantins) did not differ from respondents’ scores from the other states. The few statistically significant differences (*p* < .05) centered on one state in the North and two states in the Northeast. Respondents from Rondônia (a North state) scored lower on relational mobility than respondents from eight states (Bahia, Ceará, Espírito Santo, Maranhão, Pernambuco, Rio de Janeiro, Rio Grande do Sul, and São Paulo). Similarly, respondents from Paraiba (a Northeast state) had lower relational mobility scores than respondents from five states (Bahia, Ceará, Espírito Santo, Maranhão, Pernambuco). In contrast, respondents from Maranhão (another Northeast state) had higher relational mobility scores than respondents from five states (Mato Grosso, Paraná, and Santa Catarina; plus the differences from Paraíba and Rodônia noted above).

It is worth noting that these statistically significant differences were few, the effect sizes were small, and we did not adjust for multiple comparisons. As detailed in the main document, our findings indicate relational mobility *does not* vary across regions or states in Brazil.


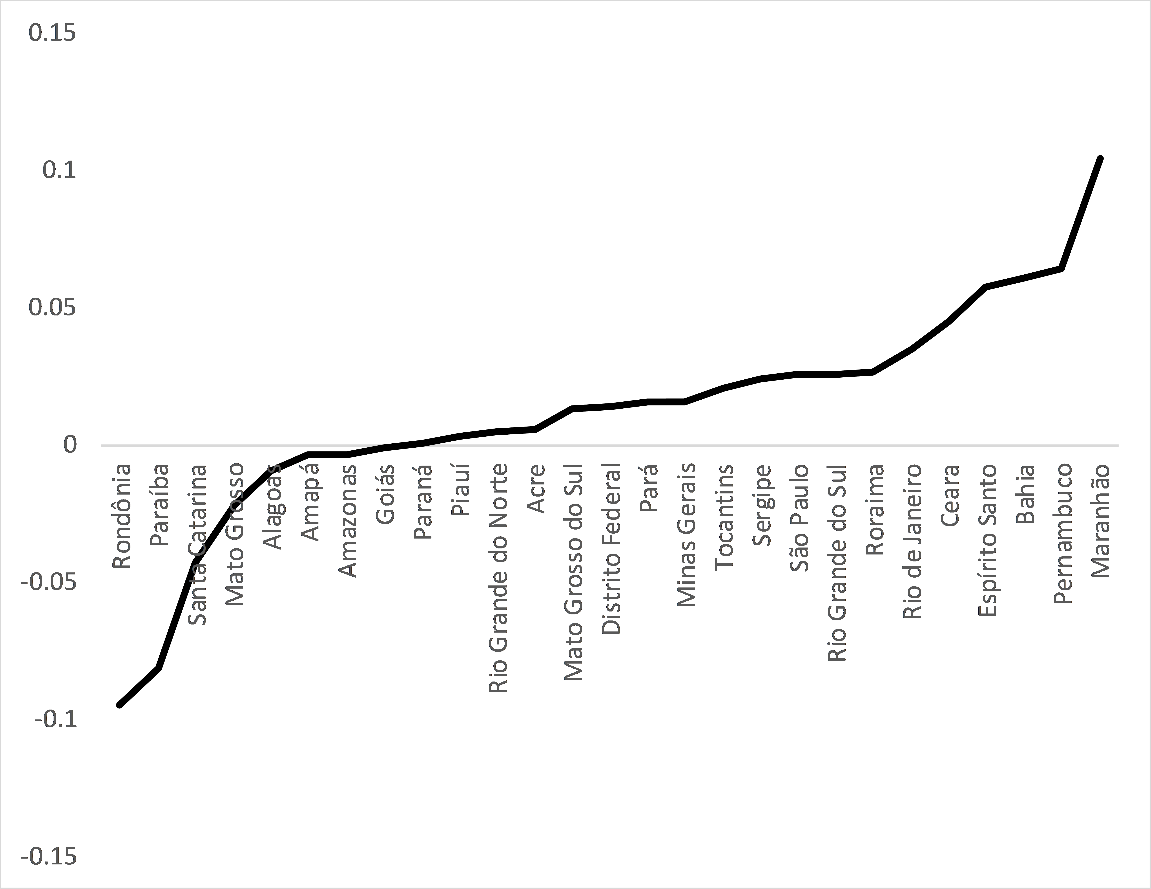


**Fig S3.** **Visual Depiction of Relational Mobility Across the Brazilian States in Study 2.**

**Relational mobility and its sub-components**

Theoretically speaking, one might expect that the *choosing* subcomponent of relational mobility might play a larger and more fundamental role than the *meeting* counterpart in shaping pro-active relational behaviours, such as intimacy and self-disclosure. This is because even if any given environment provides many opportunities to *meet* other people—like when living in a big city such as Tokyo—there should be little incentive for individuals to invest a lot of energy and resources to create and maintain relationships with those individuals unless there exists the chance that one might become friends or partners with those individuals. Likewise, if there is no risk that one’s partner might leave for better relationships—a key concept in the *choosing* subcomponent—why develop intimacy, for example (Thomson et al., 2018)? This is not to say that the *meeting* subcomponent is superfluous; theoretically, ‘meeting’ is a pre-condition for ‘choosing’ to operate. An abundance of relational choice is possible only when one has many chances to see or meet new people.

A recent study by Bahns, Lee and Crandall (2019) examined subcomponents of relational mobility. For the sake of thoroughness and following a reviewer’s request, we tested the relationship between the individual subcomponents of the relational mobility scale (i.e., the MEETING and CHOOSING factors) as well as the higher-order relational mobility factor. Results of these analyses are presented in Table S2.

Table S2. Multi-Level Analyses Predicting Interpersonal Behavior and Psychology from State-Level Relational Mobility in Study 2.

| Target | Level-1 Dependent Variable | Model ^c^ | Dependent Intercept  γ_00_  (SE) | Within-group Variance  *r*  (SE) | Between-group Variance  *u*_0_  (SE) | Level-2 Predictor |
| --- | --- | --- | --- | --- | --- | --- |
|  |  |  |  |  |  | Relational Mobility  (SE) γ_01_ |
| Close friend ^a^ | Self-disclosure | 1 | -.701^***^  (.097) | .527^***^  (.019) | <.001  (.002) | — |
|  |  | 2 (RMOB) | -.651^***^  (.096) | .526^***^  (.019) | .004  (.002) | **.261^*^**  **(.116)** |
|  |  | 2 (MEETING) | -.645^***^  (.096) | .526^***^  (.019) | .001  (.003) | **.526^*^**  **(.238)** |
|  |  | 2 (CHOOSING) | -.661^***^  (.095) | .525^***^  (.019) | .002  (.006) | .481^†^  (.283) |
|  | Similarity | 1 | -.856^***^  (.092) | 1.132^***^  (.044) | .009  (.005) | — |
|  |  | 2 (RMOB) | -.818^***^  (.097) | 1.130^***^  (.044) | .010  (.007) | **.570^*^**  **(.280)** |
|  |  | 2 (MEETING) | -.803^***^  (.092) | 1.130^***^  (.044) | .011  (.007) | .863  (.621) |
|  |  | 2 (CHOOSING) | -.829^***^  (.097) | 1.130^***^  (.044) | .009  (.006) | **1.239^*^**  **(.587)** |
|  | Intimacy | 1 | -.386^***^  (.071) | .312^***^  (.014) | .001  (.001) | — |
|  |  | 2 (RMOB) | -.386^***^  (.071) | .311^***^  (.013) | .001  (.001) | **.302^*^**  **(.121)** |
|  |  | 2 (MEETING) | -.379^***^  (.071) | .311^***^  (.013) | .002  (.001) | .343  (.217) |
|  |  | 2 (CHOOSING) | -.387^***^  (.071) | .311^***^  (.013) | .001  (.001) | **.718^**^**  **(.276)** |
| Romantic partner ^b^ | Self-disclosure | 1 | -.530^***^  (.129) | 0.790^***^  (.023) | .002  (.003) | — |
|  |  | 2 (RMOB) | -.471^***^  (.135) | 0.788^***^  (.023) | .003  (.009) | -.283  (.208) |
|  |  | 2 (MEETING) | -.483^***^  (.133) | 2.748^***^  (.115) | .003  (.026) | -.220  (.416) |
|  |  | 2 (CHOOSING) | -.473^***^  (.098) | .788^***^  (.023) | .004  (.018) | -.759^†^  (.453) |
|  | Similarity | 1 | -1.207^***^  (.329) | 2.748^***^  (.114) | .050^*^  (.020) | — |
|  |  | 2 (RMOB) | -1.088^**^  (.338) | 2.746^***^  (.115) | .042  (.026) | -.647^†^  (.362) |
|  |  | 2 (MEETING) | -1.112^**^  (.335) | 2.748^***^  (.115) | .043  (.024) | -.354  (.771) |
|  |  | 2 (CHOOSING) | -1.036^***^  (.335) | 2.746^***^  (.115) | .041  (.026) | **-1.681^*^**  **(.746)** |
|  | Intimacy | 1 | -1.283^*^  (.523) | 4.574^***^  (.193) | .024  (.016) | — |
|  |  | 2 (RMOB) | -1.223^*^  (.527) | 4.574^***^  (.192) | .023  (.038) | .376  (.435) |
|  |  | 2 (MEETING) | -1.205^*^  (.527) | 4.573^***^  (.192) | .019  (.032) | 1.902^†^  (1.081) |
|  |  | 2 (CHOOSING) | -.972^***^  (.230) | 4.575^***^  (.193) | .026  (.051) | .295  (1.264) |

^a^ *N* = 3,042, *k* = 27. ^b^ *N* = 2,196, *k* = 27. ^c^ Model 1: Unconditional means model (includes gender, age and household income as covariates at the individual level); Model 2: Regression with means-as-outcomes with relational mobility—the higher-order factor or each of the MEETING and CHOOSING factors—on the dependent variables (includes gender, age and household income as covariates at the individual level; except for the romantic partner models with the CHOOSING factor which excluded gender in order to converge). Individuals who indicated being older than 80 years old were not included in these analyses. ^***^*p* < .001, ^**^*p* < .01, ^*^*p* < .05, ^†^*p* < .10.

**References Cited in the Supplementary Material**

Newman, D. B., Schug, J., Yuki, M., Yamada, J., & Nezlek, J. B. (2018). The negative consequences of maximizing in friendship selection. *Journal of Personality and Social Psychology, 114*, 804-824. <https://doi.org/10.1037/pspp0000141>

Thomson, R., Yuki, M., Talhelm, T., Schug, J., Kito, M., Ayanian, A., Becker, J., Becker, M., Chiu, C. Y., Choi, H., Ferreira, C. M., Fülöp, M., Gul, P., Houghton-Illera, A. M., Jaosoo, M., Jong, J., Kavanagh, C., Khutkyy, D., Manzi, C., Marcinkowska, U. M., Milfont, T. L., Neto, F., von Oertzen, T., Pliskin, R., San Martin, A., Singh, P., Visserman, M. L. (2018). Relational mobility predicts social behaviors in 39 countries and is tied to historical farming and threat. *Proceedings of the National Academy of Sciences, 115*, 7521-7526. <https://doi.org/10.1073/pnas.1713191115>

Bahns, A. J., Lee, J., & Crandall, C. S. (2019). Culture and mobility determine the importance of similarity in friendship. *Journal of Cross-Cultural Psychology, 50*, 731-750. <https://doi.org/10.1177/0022022119852424>
